# Concomitant autoimmune hemolytic anemia and pulmonary embolism associated with mild COVID‐19: A case report

**DOI:** 10.1002/ccr3.4952

**Published:** 2021-10-18

**Authors:** Abdulrahman F. Al‐Mashdali, Yaser M. Ata, Mohamed A. Yassin

**Affiliations:** ^1^ Department of Internal Medicine Hamad Medical Corporation Doha Qatar; ^2^ Department of Medical Education Hamad Medical Corporation Doha Qatar; ^3^ National Center for Cancer Care and Research Department of Oncology, Hematology and BMT Section Hamad Medical Corporation Doha Qatar

**Keywords:** anemia, autoimmune hemolytic anemia, case report, COVID‐19, pulmonary embolism

## Abstract

Despite its rarity, AIHA can be associated with COVID‐19. It should be suspected in a patient with recent COVID‐19 presenting with unexplained anemia.

## Graphical abstract

Despite its rarity, autoimmune hemolytic anemia (AIHA) can be associated with COVID‐19, particularly severe cases. It should be considered in any patient with recent COVID‐19 presenting with unexplained anemia. Rarely, severe AIHA can also predispose to pulmonary embolism.

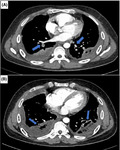

## 1 INTRODUCTION

Autoimmune hemolytic anemia (AIHA) is a rare immune‐mediated complication of COVID‐19, leading to adverse clinical outcomes. The clinician should consider AIHA in any patient with current or recent COVID‐19 developing unexplained normocytic anemia. In addition, pulmonary embolism might complicate AIHA and should also be investigated if clinically suspected.

Autoimmune hemolytic anemia (AIHA) is acquired hemolytic anemia caused by the host's immune system attacking its red blood cells.^1^ Different immunological processes are involved in the pathophysiology of AIHA, including autoantibodies, complement system, and cell‐mediated cytotoxicity.^2^ There are four types of AIHA: warm autoimmune hemolytic anemia (wAIHA), cold agglutinin disease (CAD), mixed type AIHA (mixed AIHA), and paroxysmal cold hemoglobinuria (PCH). Direct antiglobulin test (DAT)is the gold standard for the diagnosis of AIHA, basically, by detecting the attached immunoglobulins to erythrocytes.^3^ AIHA is a primary disease in around 50% of the cases, and in the remaining cases, it is associated with autoimmune diseases, lymphoproliferative disorders, infections, and solid tumors.^4^ COVID‐19 is an emerging systemic disease that has been associated with various complications, including autoimmune disorders and hypercoagulability. A limited number of AIHA cases have been reported in patients with SARS‐CoV‐2 infection, mainly associated with severe COVID‐19 cases.^5^ However, AIHA occurring with mild COVID‐19 is an extremely unusual association and might be missed if not highly considered.^6^ Herein, we report a patient who developed concurrent AIHA and pulmonary embolism (PE) after approximately two weeks of a mild COVID‐19 infection. Up to the best of our knowledge, this is the first reported case of simultaneous AIHA and PE after a mild COVID‐19.

## 2 CASE PRESENTATION

**FIGURE 1:**
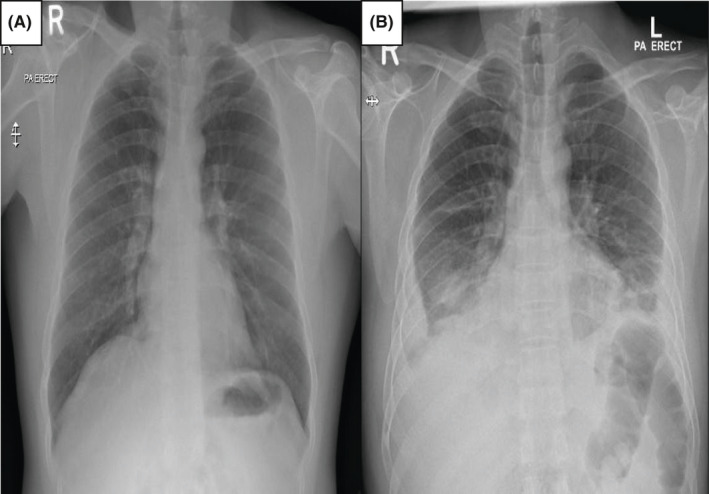
Chest X‐rays. (A) On presentation with COVID‐19 was nearly normal. (B) After three weeks of COVID‐19 showing, patchy infiltrates in the lower zones bilaterally and blunting of the costophrenic angles suggestive of pleural effusions

A 39‐year‐old Indian male with no significant past medical history presented to the emergency department (ED) on April 13, 2021, with fever and cough for three days. He was vitally stable and maintaining oxygen saturation on room air. After that, he tested positive for COVID‐19; and there was no infiltrate on the chest X‐ray (Figure 1A). Accordingly, he was diagnosed as a case of mild COVID‐19, which was treated conservatively and stayed in the quarantine facility for one week and discharged after that. He only received paracetamol and multivitamin tablets during his quarantine period.

**FIGURE 2:**
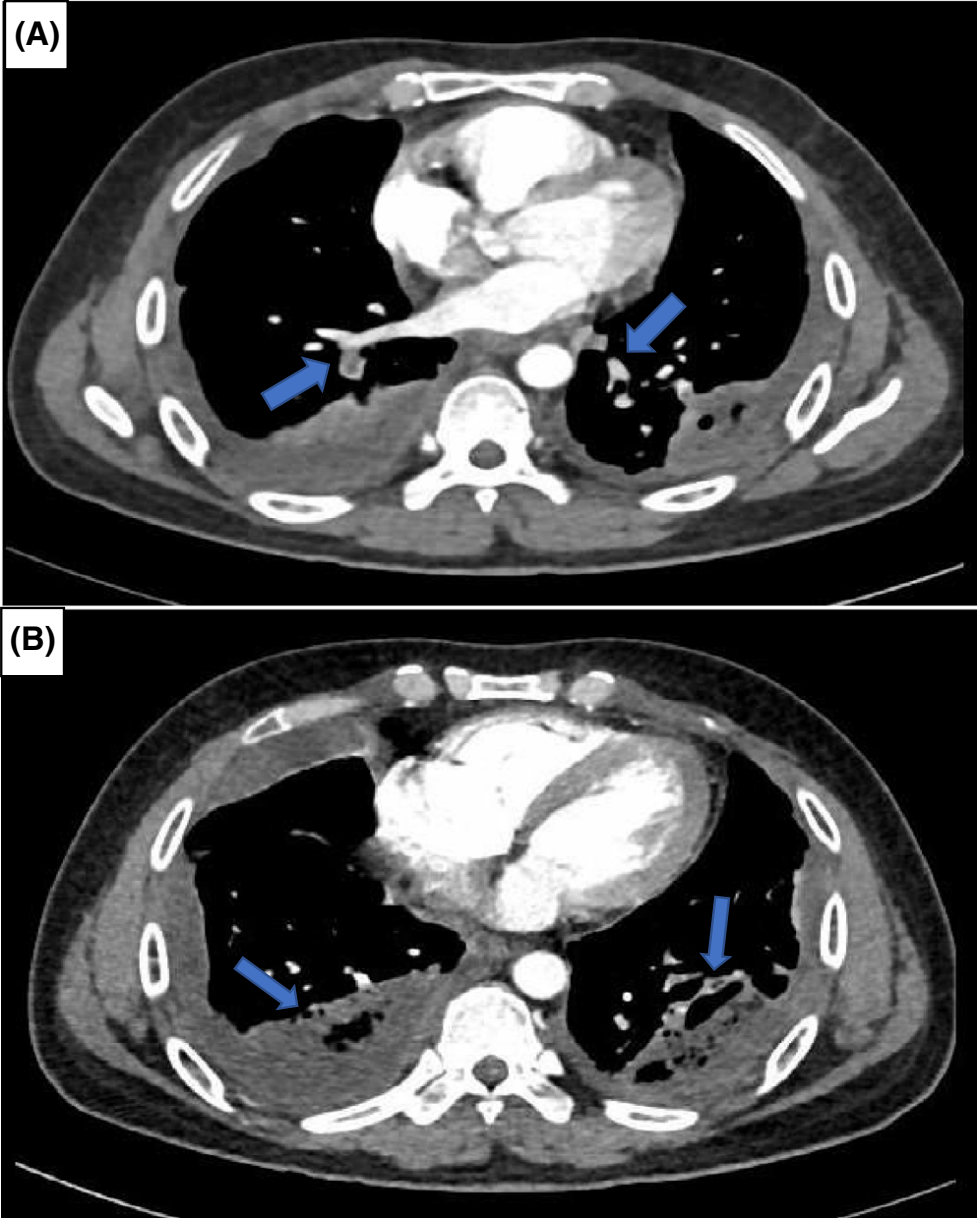
Computed tomographic pulmonary angiography (CTPA) images. (A) Showing bilateral segmental pulmonary emboli. (B) Revealing bilateral lower lobe consolidates with air bronchograms and pleural effusion

On May 6, 2021, he presented to the ED with progressive shortness of breath (SOB), cough, and chest pain for ten days. In ED, he was afebrile and had a respiratory rate of 25 breaths/min, pulse rate of 120 beats/min, blood pressure of 110/75 mmHg, and oxygen saturation of 96% on room air. Physical examination was significant for a bilateral decrease of air entry in the lung bases, and apart from that, the examination was unremarkable. Electrocardiography (ECG) revealed sinus tachycardia without features of pulmonary embolism, and CXR demonstrated patchy infiltrates in the lower zones bilaterally and blunting of the costophrenic angles suggestive of pleural effusions (Figure 2B). Repeated COVID‐19 polymerase chain reaction (PCR) was negative.

His laboratory investigations revealed a hemoglobin level of 8.2 gm/dl (his hemoglobin level was 16.4 gm/dl when he was diagnosed with COVID‐19) with normal mean corpuscular volume(MCV),elevated reticulocytes percentage (4.9%), high lactate dehydrogenase level (310 U/L), mildly elevated indirect bilirubin level (32 umol/L),normal haptoglobin,and D‐dimer of 7.2 mg/L (normal <0.45). All relevant laboratory data are shown in Table 1. He denied blackish discoloration of stool (melena), bleeding per rectum, or any bleeding from other body sites. Also, he denied a previous history of anemia or a family history of blood disorders. Accordingly, hemolytic anemia was suspected, and an extensive hemolysis workup was sent to look for the possible etiology.

**TABLE 1:** Relevant laboratory results (on admission to the hospital and upon discharge)

Laboratory parameter	On admission	Upon discharge	Reference range
WBC	11.7 × 10^3/ul	8.4 × 10^3/ul	4.0–10.0
Hgb	8.2 gm/dl	11.4 gm/dl	13.0–17.0
Platelet	571 × 10^3/ul	430 × 10^3/ul	150–400
Reticulocytes %	4.9%	2.5%	0.5–2
D‐dimer	7.2 mg/dl	1.8 mg/dl	<0.45
LDH	310 U/L	179 U/L	120–220
Haptoglobin	191 mg/dl	N/A	30–200
Indirect bilirubin	32 umol/L	N/A	<21
Creatinine	78 umol/L	N/A	70–110
Liver enzymes	Normal	Normal	‐
CRP	11 mg/L	N/A	<5

Abbreviations: CRP, C‐reactive protein; Hgb, hemoglobin; LDH, lactate dehydrogenase; N/A, not available; WBC, white blood cell.

Notably, the tachycardia did not resolve (ranging from 110 to 120), although he received a vigorous amount of intravenous fluid. So, given his history of recent COVID‐19 and current clinical findings (SOB, chest pain, tachypnea, and tachycardia), computed tomographic pulmonary angiography (CTPA) was requested to exclude pulmonary embolism. CTPA revealed bilateral pulmonary emboli, in addition to lung bases consolidation and pleural effusion (Figure 2). N‐terminal pro‐b‐type natriuretic peptide (NTproBNP) and high‐sensitivity cardiac troponin were not elevated. Echocardiography was unremarkable (no signs of right ventricular strain or dysfunction). Subsequently, the patient was started on a therapeutic dose of enoxaparin (60 mg twice daily).

Regarding hemolytic anemia investigations, peripheral blood smear demonstrated normocytic normochromic anemia with polychromasia, rouleaux, and anisopoikilocytosis, including scattered spherocytes. Serum iron profile, folate, and vitamin B12 level were within normal ranges. Glucose‐6‐phosphate dehydrogenase (G6PD) deficiency screening and hemoglobin electrophoresis (to rule out hemoglobinopathies) were normal. Testing for hepatotropic viruses, human immunodeficiency virus (HIV), Epstein‐Barr virus (EBV), cytomegalovirus (CMV), parvovirus B19, respiratory viruses, and mycoplasma pneumonia were negative. In addition, antinuclear antibody (ANA) and antineutrophil cytoplasmic antibodies (ANCA) results came negative. However, DAT was strongly positive for immunoglobulin G (IgG) and negative for both IgM and C3d. Abdominal ultrasound (AUS) was unremarkable (did not show evidence of organomegaly). Hence, the diagnosis of AIHA, most likely associated with COVID‐19 was made, and the patient started on prednisolone (60 mg daily). He was asymptomatic at day eight of the hospital stay, vitally stable (tachycardia resolved), and his hemoglobin level improved to 11.4 gm/dl. He was discharged on rivaroxaban for PE and a tapering dose of prednisolone for AIHA on day nine. Unfortunately, he lost follow‐up at the outpatient clinic because he had traveled to his home country.

## 3 DISCUSSION

SARS‐CoV 2 infection has been recently linked to the development of AIHA. Both warm AIHA (wAIHA) and cold AIHA (cAIHA) have been reported in patients with COVID‐19.^5^ Based on one review, only 20 cases of COVID‐19‐related AIHA are found in the literature. The average time between the onset of COVID‐19 symptoms to the development of AIHA is around eight days.^7^ Of note, most patients who developed AIHA had a moderate‐severe COVID‐19, and the inflammatory markers were notably elevated^7^, ^8^; however, AIHA can occur even in patients with asymptomatic COVID‐19.^6^ Moreover, a few of those patients had preexisting comorbidities, such as chronic lymphatic leukemia idiopathic thrombocytopenic purpura, or lymphomas, suggesting a possible underlying immunological phenomenon.^8^ The pathophysiology of COVID‐19 related AIHA is still not well‐understood. At the beginning of the COVID‐19 era, given that most of the AIHA events were associated with severe cases, it has been postulated that dysregulated immune system activation could be the likely cause through attacking of the red blood cells resulting in their hemolysis. However, this hypothesis did not explain the occurrence of AIHA in patients with mild or asymptomatic COVID‐19 in the absence of hyperinflammatory response. Subsequently, a new hypothesis suggests that molecular mimicry between Ankyrin 1 (an erythrocyte membrane protein) and Spike protein (SARS‐CoV‐2 surface glycoprotein) could be the culprit in AIHA development in COVID‐19 patients.^9^, ^10^ The evidence for the treatment of COVID‐19‐related AIHA is scarce and based mainly on the reported cases, although glucocorticoid therapy has been shown to be very effective and most of the cases improved dramatically after commencing glucocorticoid therapy.^5^, ^6^, ^7^, ^8^

Despite its rarity, AIHA is currently a well‐recognized risk factor for venous thromboembolism (VTE). However, this risk might be underestimated by many physicians. In a large observational study done in 2014 by Yusuf et al.^11^ it was found that the odds ratio for VTE in patients with AIHA is approximately 1.25 (95% CI: 1.05–1.49) either during or before hospitalization. Interestingly, most of the VTE events in patients with AIHA happened in outpatient's settings with the absence of classic risk factors for venous thrombosis. Also, it was observed that these thrombotic events occurred almost always during active hemolytic attack.^12^ The pathophysiology of hypercoagulability in AIHA patients is complex and multifactorial, triggered by both hemolysis and immune system effect. It has been proposed that the RBC membrane is modified through the attachment of autoantibodies, which enhancing the activation of clotting factors, especially prothrombin and factor X. Moreover, microparticles (MPs) and cell‐free hemoglobin, which are released during hemolysis, can lead to oxidative stress on vascular endothelium, enhance thrombin synthesis, and facilitate platelet aggregation. Finally, glucocorticoids therapy might play a role in the pathogenesis of VTE in patients with AIHA.^12^, ^13^, ^14^

Our patient started to develop the manifestations of AIHA almost two weeks after COVID‐19. We believe that our case is interesting from different aspects. Initially, AIHA developed after a mild COVID‐19, whereas the vast majority of COVID‐19 related AIHA occurred in severe cases. Also, our patient did not have any comorbid condition, which was found in the majority of COVID‐19 related AIHA cases, and associated with severe COVID‐19.^15^, ^16^, ^17^ Additionally, the hemolysis ensued after a lag period of two weeks, supporting the hypothesis of molecular mimicry as the most likely pathophysiology of AIHA in our patient. In addition to AIHA, concurrent multisegmental PE was diagnosed in our patient. We think that COVID‐19 is unlikely to be the predisposing factor for hypercoagulability in our patient because it was only a mild infection (the patient did not require any oxygen support) with mildly elevated inflammatory markers. Accordingly, we contemplate that AIHA (with approximately 50% drop in hemoglobin level) was the predisposing factor for PE in our patient.

## 4 CONCLUSION

COVID‐19‐related AIAH is increasingly reported in the literature. It should be highly considered in any COVID‐19 patient who develops unexplained anemia, especially if it occurs within the first few weeks of the infection. Prompt management is pivotal to decrease morbidity and mortality of such condition. Given that AIHA might occur after patient discharge from the hospital or quarantine facility, we suggest for follow‐up complete blood count within 2–4 weeks of COVID‐19 to catch this disease in the early stage. In addition, based on our case, we recommend having a lower threshold for PE diagnosis in a patient with AIHA, especially in the presence of significant dyspnea or persistent tachycardia.

## CONFLICTS OF INTEREST

The authors have no conflict of interest to declare.

## AUTHOR CONTRIBUTIONS

AFA contributed to patient care, data collection, literature review, and manuscript writing. YMA contributed to data collection and manuscript writing. MAY contributed to the final manuscript review and editing. All authors reviewed and approved the final version of the article.

## ETHICS APPROVAL

This case report was approved by the Hamad Medical Corporation's Medical Research Center (Protocol number: MRC‐04–21–581).

## CONSENT

Written informed consent was obtained from the patient for the publication of this case report.

## Data Availability

The datasets used and/or analyzed during the current study are available from the corresponding author on request.
